# Improved Outcomes in Patients with 22q11.2 Deletion Syndrome and Diagnosis of Interrupted Aortic Arch Prior to Birth Hospital Discharge, a Retrospective Study

**DOI:** 10.3390/genes14010062

**Published:** 2022-12-24

**Authors:** Hayley A. Ron, Terrence Blaine Crowley, Yichuan Liu, Marta Unolt, Erica Schindewolf, Julie Moldenhauer, Jack Rychik, Elizabeth Goldmuntz, Beverly S. Emanuel, Douglas Ryba, James William Gaynor, Elaine H. Zackai, Hakon Hakonarson, Donna M. McDonald-McGinn

**Affiliations:** 1 Children’s Hospital of Philadelphia, Philadelphia, PA 19104, USA; 2 Department of Pediatrics, Perelman School of Medicine, University of Pennsylvania, Philadelphia, PA 19104, USA; 3 Division of Cardiology, Ospedale Bambino Gesu and Sapienza University of Rome, Viale del Policlinico 155, 00161 Rome, Italy; 4 Department of Human Biology and Medical Genetics, Sapienza University, Viale del Policlinico 155, 00161 Rome, Italy

**Keywords:** interrupted aortic arch, type B interrupted aortic arch, 22q11.2 deletion syndrome, congenital heart disease, critical congenital heart disease screening, prenatal diagnosis

## Abstract

Interruption of the aortic arch (IAA) is a rare but life-threatening congenital heart defect if not corrected in the neonatal period. IAA type B is highly correlated with 22q11.2 deletion syndrome (22q11.2DS); approximately 50% of patients with IAA type B also have 22q11.2DS (Peyvandi et al.; Goldmuntz et al.). Early identification and repair of IAA can prevent severe morbidity and death. However, IAA is challenging to identify prenatally, or even in the neonatal period. In this study, we examined infants with IAA, diagnosed during pregnancy and prior to discharge (PPTD) from the birth hospital vs. those diagnosed following discharge (FD) from the newborn nursery. Our goals were to determine: (1) if early diagnosis improved outcomes; and (2) if patients with IAA and without 22q11.2DS had similar outcomes. In total, 135 patients with a diagnosis of 22q11.2DS and IAA were ascertained through the 22q and You Center at the Children’s Hospital of Philadelphia (CHOP). The examined outcomes included: timing of diagnosis; age at diagnosis (days); hospital length of stay (LOS); duration of intensive care unit (ICU) stay; mechanical ventilation (days); duration of inotrope administration (days); year of surgical intervention; birth hospital trauma level; and overall morbidity. These outcomes were then compared with 40 CHOP patients with IAA but without 22q11.2DS. The results revealed that the PPTD neonates had fewer days of intubation, inotrope administration, and hospital LOS when compared to the FD group. The outcomes between deleted and non-deleted individuals with IAA differed significantly, in terms of the LOS (40 vs. 39 days) and time in ICU (28 vs. 24 days), respectively. These results support the early detection of 22q11.2DS via prenatal screening/diagnostics/newborn screening, as IAA can evade routine prenatal ultrasound and postnatal pulse oximetry. However, as previously reported in patients with 22q11.2DS and congenital heart disease (CHD), patients with 22q11.2DS tend to fare poorer compared to non-deleted neonates with IAA.

## 1. Background

### 1.1. Anatomy of IAA

Interruption of the aortic arch (IAA) is a rare but serious congenital heart defect. It is characterized by complete anatomic discontinuity or by an atretic fibrous strand between the aortic arch and the descending aorta. It has been classified into three types. Type B is the most common and consists of interruption between the left common carotid and the left subclavian arteries. Interruption distal to the left subclavian artery comprises Type A. The least frequent interruption is between the innominate artery and the left carotid artery, known as type C (Perloff and Marelli, 2012) [1]. 

IAA Type B rarely occurs in isolation and is most frequently seen with a patent ductus arteriosus (PDA) and a ventricular septal defect (VSD) or atrial septal defect (ASD). The neonate’s survival depends on the presence and patency of these structures (Takashina et al., 1972) [2]. When the ductus closes following birth, blood supply to the lower body is abolished and can lead to profound ischemia. Furthermore, all pulmonary blood flow is directed to the overloaded lungs (Schumacher et al., 1986) [3].

When ductal-dependent cardiac defects such as IAA are identified prenatally, the standard of care is to administer prostaglandins at birth to maintain the patency of the ductus until surgery can be undertaken. Additionally, the infant can be delivered at (or immediately transferred to) a higher-level facility equipped to manage the condition. 

### 1.2. Prevalence and Implication of 22q11.2DS in IAA

Approximately 60–80% of patients with 22q11.2DS have an associated cardiac anomaly (Campbell et al., 2018) [4]. Specifically, IAA comprises about 11% of cardiac defects in our 22q11.2DS patient population at the Children’s Hospital of Philadelphia (CHOP) in Philadelphia, PA, USA. 

The diagnosis of aortic arch anomalies should always prompt investigation into a chromosome 22q11.2 deletion. It is estimated that approximately 50% of patients with IAA have 22q11.2DS as the etiology of the defect (Goldmuntz et al., 1998; Peyvandi et al., 2013; Volpe et al., 2002) [5,6,7]. Rarely, IAA type A and type C are associated with 22q11.2DS (Lindsay et al., 1995; Takahashi et al., 1999) [8,9]. 

The diagnosis of 22q11.2DS may also be useful surrounding surgical repair and planning. Neonates with congenital heart disease, including IAA, who underwent surgical repair in the neonatal period had a longer length of hospital stay, duration of intubation, and duration of intensive care unit (ICU) stay if they had 22q11.DS, compared to patients without the deletion (O’Byrne et al., 2014) [10].

### 1.3. Prenatal Diagnosis of IAA

The prenatal diagnosis of IAA, while challenging, is improving. Between 1988 and 2009, one study found that over this 21-year period, a total of 24% of patients born with an IAA were detected prenatally. The frequency of prenatal diagnosis increased from 11% in the first seven years of the study to 43% in the final seven years of the study (Vogel et al., 2010) [11]. In Germany, between 1994 and 2010, another study found that 8 of 28 patients (29%) with IAA were diagnosed prenatally. All prenatally detected cases occurred between 2005 and 2010, in the final five years of their study (Axt-Fliedner et al., 2011) [12]. The improved detection rate over time is likely the result of several factors. including improved imaging quality, experience of the sonographer, increased awareness, and more frequent referrals for fetal echocardiograms.

The essential elements of the fetal anatomy scan for a low-risk, uncomplicated pregnancy, dictated by the American College of Obstetricians and Gynecologists (ACOG) and the American Institute of Ultrasound in Medicine (AIUM), include visualization of a 4-chamber view and the left and right outflow tracts (Practice Bulletin 175, 2016) [13], which can appear normal in a fetus with a conotruncal defect (Vogel et al., 2010) [11]. A more detailed anatomic examination is only indicated when there is an increased risk of an anomaly based on the history, abnormal laboratory findings, or concerns raised on routine examination. There may also be difficulty differentiating between the aortic arch and the ductal arch on prenatal ultrasounds (Vogel et al., 2010) [11]. 

While the defect itself may not be visualized on a routine anatomy scan, there may be clues in the other examined structures. There may be a discrepancy in the diameter of the great vessels in fetuses with IAA (Axt-Fliedner et al., 2011; Vogel et al., 2010; Volpe et al., 2002) [7,11,12]. In addition, the aortic valve may be significantly smaller than the pulmonary valve in affected fetuses (Vogel et al., 2010) [11]. In one study, a right ventricle-left ventricle (RV/LV) size discrepancy was consistently identified with IAA; this finding should, therefore, direct the sonographer to carefully visualize the great vessels (Volpe et al.) [7]. Other studies contradicted this, however, stating that the unrestrictive VSD allows for equally sized ventricles (Vogel, Axt-Fliedner) [11,12]. IAA almost always occurs with a posterior malalignment VSD. The presence of a VSD of any kind, but particularly of conotruncal VSDs, should therefore prompt further characterization of cardiac structures.

### 1.4. Deletion Size

The diagnosis of 22q11.2DS is made by identifying a heterozygous deletion at chromosome 22q11.2. Fluorescence in situ hybridization (FISH) analysis for 22q11.2DS began in 1991 in the UK and at CHOP, respectively (Scambler et al., 1991; Driscoll et al., 1992) [14,15]. Targeted deletion analysis can now be performed using FISH or multiplex ligation probe amplification (MLPA). Chromosomal microarray also identifies the deletion and, similarly to MLPA, provides information regarding the size of the deletion. Moreover, as microarrays are genome-wide (not specific to chromosome 22q11.2) detection of the deletion can occur even in those individuals where this is not an a priori elevated index of suspicion. The majority of patients with 22q11.2 DS (about 85%) harbor a de novo heterozygous deletion of approximately 2.54 Mb in length spanning chromosome 22q11.2 low-copy repeats (LCRs) A to D. Only about 5% of individuals harbor a heterozygous nested 1.5 Mb deletion from LCR22A to LCR22B, and about 2% have a deletion extending from LCR22A to LCR22C. Another 5% have a heterozygous nested deletions extending from LCR22B-LCR22D or LCR22C-LCR22D. These latter deletions (LCR22B-LCR22D and LCR22C-LCR22D) would be missed by FISH alone as the probes are located between LCR22A- LCR22B (McDonald-McGinn et al., 2015.) [16]. 

Illustrative Case: A female 2890 gm product of a full term, uncomplicated gestation was born in 1992 by SVD to a then G1P0 28-year-old mother and admitted to the newborn nursery. On day of life (DOL) 3, the infant’s nurse noted an increased heart rate, perioral cyanosis and decreased responsiveness. The pediatrician was notified, and an EKG and chest x-ray were obtained. The EKG was reportedly normal. The x-ray showed slightly prominent pulmonary vascularity and a normal heart size. On DOL 4, the infant was discharged from the hospital. On DOL 5 and 7, the patient was seen by the pediatrician, for what appeared to be excessive weight gain. The parents continued to have concerns and by DOL 8 the infant was taken to the emergency room where she suffered a cardiopulmonary arrest. The patient was treated for severe multi-organ failure and required urgent cardiac repair. She suffered a tibial growth plate injury as a result of the intraosseous needle insertion required during her resuscitation, that several surgeries were unable to correct. She subsequently had poor growth on all parameters (length, weight and head circumference) and global developmental delay. 

## 2. Materials and Methods

A retrospective cohort design was chosen, examining patients with a laboratory confirmed chromosome 22q11.2 deletion with IAA and those with IAA without a 22q11.2 deletion. The data were collected through a review of hospital medical records. Overall, 135 patients were selected who had a diagnosis of 22q11.2DS and IAA Type A, B or C. Demographic and relevant clinical information from the index hospitalization were abstracted. The 22q11.2DS cohort was divided into two groups based on timing of diagnosis: PPTD vs. FD. Prenatal or postnatal diagnosis of 22q11.2DS was ascertained.

Outcomes of interest included hospital length of stay (LOS), duration of ICU stay, duration of intubation, and duration of inotropic medication usage for the patients who survived the index hospitalization. For those who were discharged home without a cardiac diagnosis from their birth hospital, age at time of diagnosis and the presence or absence of shock at time of presentation was determined. Shock was determined by diagnosis in the medical record by the emergency room or admitting physician or extrapolated by documentation of: severe hypotension, tachycardia, lethargy or unresponsiveness, acidosis, signs of end organ damage, and/or severe cardiac dysfunction. Neonatal deaths were considered as those who did not survive the index hospitalization. Death during a subsequent hospitalization was considered separately in the analysis. 

CHD was defined as: moderate, complex, Type A, or Type C. Moderate was defined as Type B IAA with any of the typical associated findings: VSD, ASD, bicuspid aortic valve, aortic stenosis or subaortic stenosis, PDA, PFO, aberrant right subclavian artery. Complex was defined as Type B IAA with other anomalies including truncus arteriosus, hypoplastic left heart, double outlet right ventricle, double aortic arch, and transposition of the great arteries. Type A includes patients with Type A IAA. Type C includes one patient with Type C IAA. 

Additional variables included birth weight, gestational age, inherited deletions vs. de novo deletions, deletion size and decade of birth (DOB) which was divided into 1981–1990, 1991–2000 and so on. 

LOS was defined as the number of days in the hospital for the index hospitalization. Duration of intensive care was defined as the number of days between admission to the neonatal intensive care unit or cardiac intensive care unit preoperatively until transfer (or discharge) from intensive care. Duration of inotrope usage was defined as the total number of days that inotropic medication was required during the index hospitalization. Duration of intubation refers to the total number of days mechanical ventilation was used during the index hospitalization. 

## 3. Results

### 3.1. Cohort

The present study includes 135 patients with 22q11.2DS. Specifically, 131 with IAA type B, three with IAA type A and one with IAA type C. Eight infants were born before 36 weeks gestation and were considered separately. Four patients died during the index hospitalization and were considered separately.

### 3.2. Deletion Size

Most patients in whom deletion sizing was known had a standard LCR22A-LCR22D deletion (90%). Specifically, 78 were A-D, five were A-B, three were A-C, and one was B-D. Deletion sizing was not conducted for 48 patients. The individuals with IAA who were born prior to the ability to perform FISH, in 1991, were diagnosed with the chromosome 22q11.2 deletion syndrome sometime thereafter. 

### 3.3. Prenatal Diagnosis

**Figure 1:**
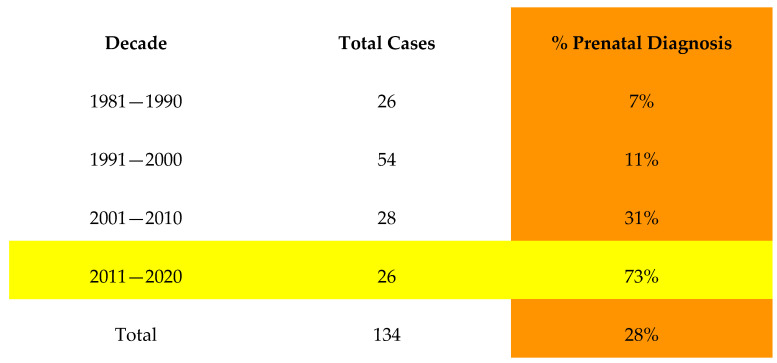
Demonstrating increasing prenatal detection rate of IAA by decade.

Between the years 1980 and 2020, there were 38/134 (28%) patients with 22q11.2DS who had a prenatal diagnosis of CHD (Figure 1). Fifty-eight percent of cases were detected in the last seven years of the study, between 2013 and 2020. In one case, there was prenatal detection of the VSD only and an echocardiogram performed shortly after birth identified IAA. Of note, there were 20 patients overall with a prenatal diagnosis of 22q11.2DS, and in each of those cases, the cardiac diagnosis prompted the genetic testing. 

### 3.4. Postnatal Detection after Birth Hospital Discharge

There was a total of 96 postnatal diagnoses of IAA. Of these, 26 neonates (27%) were discharged from the newborn nursery without a diagnosis of CHD. Of these, nine were born in the year 2011 or later, following the implementation of CCHD newborn screening. The average age of CHD diagnosis was 6.3 days for patients who were discharged prior to receiving a cardiac diagnosis with a range of two to 17 days of age. At least nine of these patients (35%) presented as being in shock, and account for two of the four index hospitalization deaths in this cohort. 

### 3.5. Level of Care of Birth Hospital

**Figure 2:**
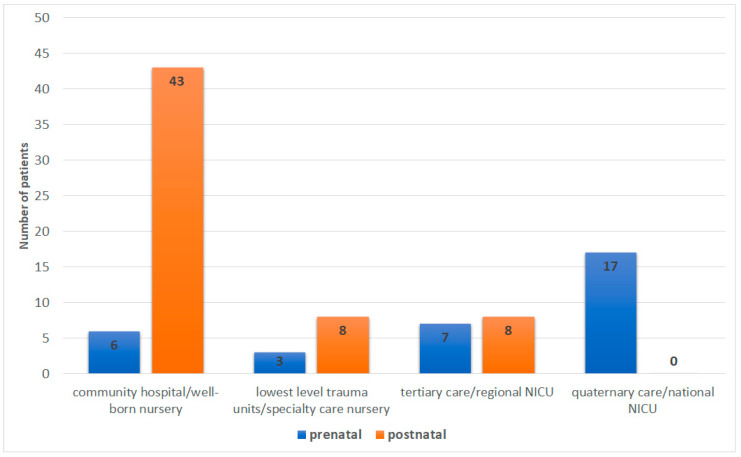
Difference in outcomes between prenatal and postnatal IAA detection. See Supplementary File S1 for complete details of the analysis.

For this analysis, the cohort was divided into cardiac diagnoses made during the pregnancy (prenatal) vs. after delivery (postnatal). Hospital trauma level was ascertained for each known birth hospital [17]. There were data available regarding birth hospital trauma levels for 92 patients. Delivery at a quaternary center occurred exclusively in those with a prenatal diagnosis (17/17). Of the postnatal diagnoses, the majority were born at a community hospital (43/59 or 73%) (Figure 2). 

### 3.6. Length of Hospital Stay

The mean LOS (days) for PPTD vs. FD is 33.7 vs. 35.2 days. There was complete information for factor analysis on 59 patients, which revealed that birth weight and timing of diagnosis were the most significant impactors of LOS.

### 3.7. Length of ICU Stay 

**Figure 3:**
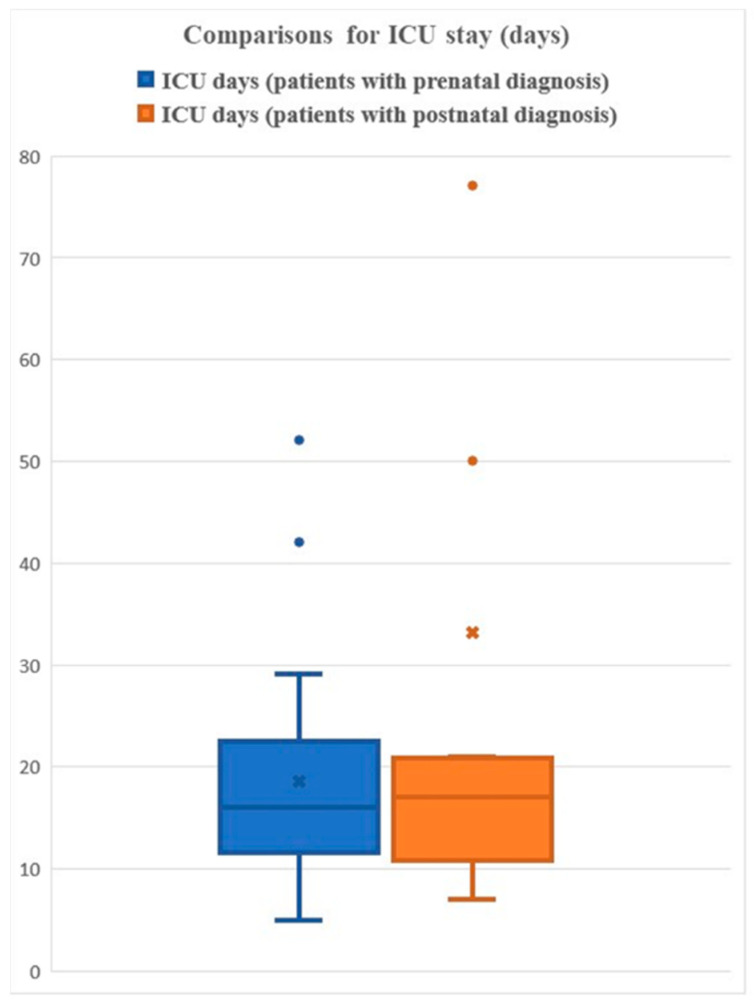
Length of ICU stay.

The mean ICU (days) for PPTD vs. FD is 18.5 vs. 33.1 days (Figure 3). There was complete information for factor analysis on 37 patients, which revealed that the timing of the diagnosis was the most significant impactor of ICU time.

### 3.8. Duration of Intubation

The mean intubation time (days) for PPTD vs. FD is 4.5 vs. 4.3. There was complete information for factor analysis on 29 patients, which revealed that gestational age and the timing of the diagnosis are the most significant impactors of intubation times.

### 3.9. Duration of Inotrope Usage

The mean inotrope administration (days) for PPTD vs. FD is 2.7 vs. 3.5. There was complete information for factor analysis on 26 patients, which revealed that gestational age and the timing of the diagnosis are the most significant impactors of inotrope administration. 

### 3.10. Shock

The presence of shock was determined for the infants with postnatal cardiac diagnoses only (*n* = 25). There was complete information for factor analysis on 18 patients. Two factors were generated, where factor 1 (DOB, Gestational Age, Discharged) explains 31.5% of whether shock developed. The DOB group weight is −0.447, implying a negative contribution to the development of shock. The weight of “discharged” is 0.976, implying a strong positive contribution to shock.

### 3.11. Mortality

Overall, there were eight deaths in total. 

Four occurred during the index hospitalization, including: a female infant born prematurely at 34 weeks, following a prenatal diagnosis of IAA who died post-operatively at 16 days of life; a male infant who inherited the 22q11.2 deletion from his father and was diagnosed with a complex cardiac lesion prenatally; a third infant presented to the hospital in shock at four days of life and died at two months of age due to complications of sepsis; and a female infant who was discharged from the newborn nursery, diagnosed with IAA at six days of age when she presented in shock, and died at nine days of age following unsuccessful efforts to resuscitate.

Four additional patients died on subsequent hospitalizations, including: two who died in infancy (less than 12 months of age) and two who died in later childhood/adolescence. One of the infants died secondary to complications of an upper respiratory infection at ten months of age; he had inherited the deletion from an affected mother and had two affected siblings, one of whom also died in infancy. The second infant died at seven months of age on a hospital admission for cardiac catheterization. 

### 3.12. Comparison to Non-Deleted Patients with IAA

#### 3.12.1. ICU

Length of ICU stay was available for 50 patients with IAA and 22q11.2DS and 40 patients with IAA without 22q11.2DS. Statistical comparisons between the two groups were performed using the Wilcoxon rank sum test, with a *p* value = 0.048. The mean ICU stay is 28 vs. 24 days for deleted vs. non-deleted patients. 

#### 3.12.2. LOS

**Figure 4:**
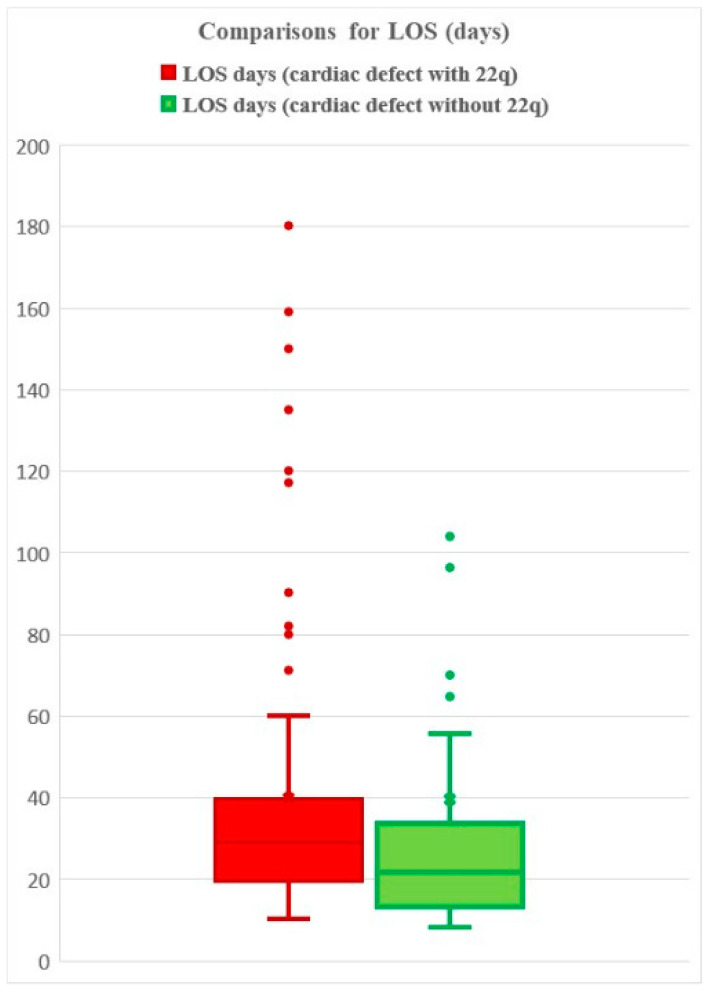
Length of Hospital Stay for those patients with IAA with and without 22q11.2DS.

LOS was available for 85 patients with IAA and 22q11.2DS and 40 patients with IAA and without 22q11.2DS. Statistical comparisons were performed using the Wilcoxon rank sum test, with a *p* value 1.8 × 10^−7^. The mean LOS is 40 vs. 39 days, respectively (Figure 4). Of note, the higher average in the control group includes two outliers (224, 292 days). When removing the outliers and reanalyzing the data without the outliers, we observed a mean of 40 vs. 28 days for deleted v. non-deleted patients. 

### 3.13. Parental Deletion Status

There were 14 patients with 22q11.2DS found to be inherited: eight paternal and six maternal. Only two of the maternal deletions and one of the paternal deletions were known prior to the pregnancy. Importantly, of the remaining 11 cases, the diagnosis of the parent was made after the child was diagnosed with 22q11DS, even when parents in hindsight were found to have clinical features associated with 22q11.2DS, such as CHD and cleft palate. 

Two patients in the overall 22q11.2DS cohort were adopted and therefore testing of the biological parents was not possible.

## 4. Discussion

Here, we describe the largest cohort, to date, reporting on patients with IAA and 22q11.2DS. Birth weight and timing of diagnosis were the most significant impactors of LOS; timing of the diagnosis was the most significant impactor of ICU time; gestational age and timing of diagnosis are the most significant impactors of intubation time; gestational age and timing of diagnosis are the most significant impactors of inotrope administration

On average, the LOS, length of ICU stay, and length of inotrope administration were shorter for the PPTD group compared to the FD group. Mean LOS and ICU measurements were longer for deleted compared to non-deleted individuals with IAA; while the means were close, the distributions of LOS and ICU times were significantly different based on nonparametric statistical tests between deleted and non-deleted individuals. 

Shock was a unique parameter because the infants with a prenatal IAA diagnosis were started on prostaglandins at birth and delivered at higher level centers. Consequently, we focused our comparison on postnatal diagnoses that occurred prior to birth hospital discharge vs. following discharge. We found that patients born in later decades were less likely to develop shock and those who were diagnosed after discharge from the birth hospital were very likely to develop shock.

The mortality in this IAA cohort, overall, was 6%. For comparison, the overall mortality rate in patients with 22q11.2DS is approximately 4–5%, at an average age of five months (Campbell et al., 2018; Mcdonald-Mcginn et al., 2001) [4,18]

Part of the challenge in the early diagnosis of IAA is that the affected neonate may be acyanotic and well appearing after birth while the ductus is open and pulmonary blood flow is preserved. Femoral pulses may be present prior to the closure of the ductus due to the blood flow to the descending aorta. Murmurs may be minimal or absent altogether (Roberts et al., 1962) [19]. There is often a failure to detect differential cyanosis of the limbs, or a marked difference in oxygen saturation, due to intracardiac shunts and collateral circulation (Higgins et al., 1977) [20]. When the ductus closes, the neonate acutely develops congestive heart failure as perfusion to the lower body is severely compromised and is dependent on collateral communications between the aortic systems. This hypoperfusion leads to end organ injury and profound acidosis. Lethargy, decreased responsiveness and seizures may develop in the untreated neonate (Jonas, 2015) [21]. 

To estimate the number of infants detected and missed via critical congenital heart disease (CCHD) screening, which was added to the Recommended Uniform Screening Panel (RUSP) in 2011, a simulation model was developed. This study approximated that 900 infants with non-syndromic CHD were likely to be detected through universal CCHD screening. However, an approximately equal number of cases would likely be missed. Of the cases missed, those with COA/IAA or TOF were the most likely to be false negatives (Ailes et al., 2015) [22]. While this study was intended for non-syndromic infants, 22q11.2 deletion is often not easily recognized at birth as dysmorphic features may be subtle or absent altogether. 

The diagnosis of 22q11.2DS in a patient has important implications for the family as the patient will likely need increased medical surveillance, increased medical interventions, and accommodations in school settings, depending on the severity of their manifestations. Furthermore, when the deletion is found to be inherited, it often helps to explain findings in the parent previously manifesting in childhood e.g., learning difficulties, short stature, CHD, cleft palate, etc. and has important genetic counseling and reproductive implications as the parent with 22q11.2DS has a 50% chance of passing on the deletion with each pregnancy. In one case in our cohort, in which a newborn, and subsequently the mother, was diagnosed postnatally, the couple elected to undergo in-vitro fertilization with preimplantation genetic diagnosis with their second pregnancy. 

There are important considerations and limitations of our study to be considered. First, we had complete data on a subset of patients who were admitted to our institution, or those who came to their outpatient genetics consultation with complete hospital records from their index hospitalization. Second, we analyzed a cohort spanning a time period of over four decades. During this time, prenatal diagnosis has improved, surgical techniques have changed, and overall medical care has evolved. The variable of time, therefore, has an important effect on the outcomes that cannot be completely quantified. 

Despite these limitations, these data provide important and actionable messages. Prenatal and early neonatal diagnosis of 22q11.2DS allows for early and thorough cardiac screening. Although prenatal detection is improving, IAA may still evade the routine anatomy scan, and CCHD screening does not reliably detect the defect prior to hospital discharge. 

The live-birth prevalence of 22q11.2 DS is estimated to be 1 in 2148 (4.7 per 10,000) (Blagojevic et al., 2021) [23], making it the most common of rare diseases. Early detection can be achieved through non-invasive prenatal testing, performed as early as ten weeks into gestation. This would prompt referral for definitive genetic testing, fetal echocardiogram and delivery at a high-level facility. Given prenatal diagnosis is not possible for, or desired by, every expectant parent (for a variety of reasons not limited to socioeconomic factors, differences in provider knowledge and familiarity with genetic testing, and insurance coverage), newborn screening, developed for 22q11.2DS in 2012 using qfPCR but not yet adopted, would allow for universal access to screening. This would direct screening, not only for cardiac defects, but also for other well-known associated complications of 22q11.2DS, such as hypocalcemia, immune dysfunction, and feeding difficulties that can pose a significant risk to the neonate, not to mention the opportunity for early interventions and genetic counseling.

## Data Availability

Not applicable.

## Supplementary Materials

The following are available online at https://www.mdpi.com/article/10.3390/genes14010062/s1, File S1: Statistical Analysis [24].
