# Supplementary material for: Improved Outcomes in Patients with 22q11.2 Deletion Syndrome and Diagnosis of Interrupted Aortic Arch Prior to Birth Hospital Discharge, a Retrospective Study
Source: Genes (Basel). 2022 Dec 24;14(1):62. doi: 10.3390/genes14010062 (PMC9859187; doi:10.3390/genes14010062)
Supplement: Supplementary file 1 [file genes-14-00062-s001.zip › genes-2000876-supplementary.pdf]

Factor analysis was applied for patients who were born after 36 weeks. We explored the associated weights of specific variables, including moderate vs complex cardiac defect, gestational age, birth weight (grams), cardiac diagnosis made prenatal and prior to discharge (PPTD) vs following discharge (FD), decade of birth, with important clinical measurements including length of hospital stay (LOS), times spent in intensive care unit (ICU), days of inotrope administration, shock and death. DOB were categorized into 10-year periods, for example patients born from 1980-1989 were grouped into A, 1990-1999 grouped into B etc. Factor analysis is a data driven analysis, therefore there is no assumption of independence of variables as in other regression models. Rather, it merges independent variables together to form a "factor" and provides the weight of the factor that explains the data variations. The factor analysis methods were based on the Scikit-learn package in Python language (version 0.21.3).

Reference
Pedregosa FV, G., Gramfort, A., Michel, V., Thirion, B., Grisel, O., Blondel, M., Prettenhofer, P., Weiss, R., Dubourg, V., Vanderplas, J., Passos, A., Cournapeau, D., Brucher, M. and Perrot, M., Duchesnay, E.: Scikit-learn: Machine Learning in Python. *Journal of Machine Learning Research* 2011, **12**:2825-2830.

Report and interpretation of the factor analysis results.
1) Patients born before 36 weeks (<36 weeks) were removed
2) Patient deceased column = 1 were removed (died in the index hospitalization)
3) DOB were categorized into 10 years period, for example patients born from 1980-1989 grouped to A, 1990-1999 grouped to B, etc.

Y variables are: LOS, ICU time, Shock, Inotropes, Deceased
X variables are: Complex_OR_Moderate, Gestational Age, Birth Weight (Grams), Prenatal and Prior to Discharge_OR_Following Discharge (PPTD_OR_FD), DOB groups

For each Y variable, we performed factor analysis based on five X variables.

LOS: in total 59 patients have complete information for factor analysis, including 32 PPTD. The mean LOS (days) for PPTD vs FD is 33.69 vs 35.22; median is 29.5 vs 29 days.

Two factors were generated, where factor 1 (Gestational Age, Birth Weight) explain 23.4% of LOS variations, where Complex_OR_Moderate weight is 0.061, DOB group weight is -0.109, Gestational Age weight is 0.401, Birth Weight is 0.993, and PPTD_OR_FD weight is -0.088; factor 2

(Complex_OR_Moderate, DOB groups, PPTD_OR_FD) explain 22.8% of the variations, where Complex_OR_Moderate weight is 0.227, DOB group weight is 0.561, Gestational Age weight is -0.07, Birth Weight is 0.121, and PPTD_OR_FD is 0.867. The interpretation is that **Birth Weight and PPTD_OR_FD are the strongest impactors of LOS.**

ICU time: In total 37 patients have complete information for factor analysis, including 21 PPTD. The mean ICU (days) for PPTD vs. FD is 18.5 vs 33.1; median is 16 vs 17 days.
Two factors were generated, where factor 1 (Complex_OR_Moderate, DOB groups, PPTD_OR_FD) explain 26.1% of ICU variations, where Complex_OR_Moderate weight is 0.379, DOB group weight is 0.507, Gestational Age weight is -0.283, Birth Weight is 0.131, and PPTD_OR_FD weight is 0.898; factor 2 (Gestational Age, Birth Weight) explain 20.9% of the variations, where Complex_OR_Moderate weight is 0.056, DOB group weight is -0.042, Gestational Age weight is -0.228, Birth Weight is 0.991, and PPTD_OR_FD weight is 0.073. The interpretation of this analysis is that **"PPTD_OR_FD " is the strongest impactor of ICU time.**

Intubation, in total 29 patients have complete information for factor analysis, including 26 prenatal. The mean intubation (days) for pre vs post is 4.5 vs 4.3; median is 2 vs 4 days.
Two factors were generated, where factor 1 (Gestational Age, Birth Weight, PPTD_OR_FD) explain 21.9% of LOS variations, where Complex_OR_Moderate weight is -0.029, DOB group weight is 0.086, Gestational Age weight is 0.988, Birth Weight is 0.243, and PPTD_OR_FD weight is 0.233; factor 2 (DOB groups, PPTD_OR_FD) explain 19.2% of the variations, where Complex_OR_Moderate weight is 0.188, DOB group weight is 0.568, Gestational Age weight is -0.143, Birth Weight is 0.135, and PPTD_OR_FD is 0.751. The interpretation of this analysis is that **Gestational Age and PPTD_OR_FD diagnosis are the strongest impactors of intubation time.**

Inotropes: 26 patients have complete information for factor analysis, including 20 PPTD. The mean time of inotropes for pre vs post is 2.7 vs 3.5; median is 2 vs 2.5.
Two factors were generated where factor 1 (DOB group, Birth Weight, PPTD_OR_FD) explain 24.1% of Inotropes times, where Complex_OR_Moderate weight is 0.309, DOB group weight is 0.497, Gestational Age weight is -0.021, Birth Weight is 0.401, and PPTD_OR_FD weight is 0.838; factor 2 (Gestational Age) explain 20.5% of the variations, where Complex_OR_Moderate weight is -0.151, DOB group weight is 0.0013, Gestational Age contributes weight is 0.998, Birth Weight is 0.041, and PPTD_OR_FD weight is -0.07. The interpretation of this analysis is that **Gestational Age and PPTD_OR_FD are the strongest impactors of inotrope usage.**

Shock: Patients with a postnatal cardiac diagnosis only were considered and the timing of the diagnosis as prior to discharge vs. following discharge was considered. Sixteen patients have complete information for factor analysis. Two factors were generated, where factor 1 (DOB_group, Gestational Age, discharged_OR_not discharged) explain 31.5% of whether Shock represented, where DOB group weight is -0.447, Gestational Age weight is 0.324, Birth Weight is -0.029, and discharged_OR_not discharged weight is 0.976; factor 2 (Birth weight) explain 26.6% of the variations, where DOB group weight is 0.162, Gestational Age weight is -0.016, Birth Weight is 0.997, and discharged_OR_not discharged weight is 0.211. **The data suggests that the later the patients were born (i.e. a more recent decade of birth), the less likely they were to develop shock AND if they were discharged from the hospital without a diagnosis, they were very likely to develop shock.**

Deceased: Due to the small number of deceased infants factor analysis cannot be completed. These cases are reviewed descriptively in the study.
